# Preoperative nutritional status and its association with adverse events following open abdominal aortic aneurysm repair

**DOI:** 10.3389/fnut.2026.1776781

**Published:** 2026-04-16

**Authors:** Eliza Russu, Patrik Buzgău, Constantin Claudiu Ciucanu, Alexandru Mureșan, Răzvan Cătălin Son, Agatha Maria Ilioniu, Réka Bartus, Eliza-Mihaela Arbănași, Vasile Bogdan Halațiu, Raluca Niculescu, Adrian Vasile Mureșan, Emil-Marian Arbănași

**Affiliations:** 1 Department of Vascular Surgery, George Emil Palade University of Medicine, Pharmacy, Science, and Technology of Targu Mures, Targu Mures, Romania; 2 Clinic of Vascular Surgery, Mures County Emergency Hospital, Targu Mures, Romania; 3 Regenerative Medicine Laboratory, Centre for Advanced Medical and Pharmaceutical Research (CCAMF), George Emil Palade University of Medicine, Pharmacy, Science and Technology of Targu Mures, Targu Mures, Romania; 4 Department of Anatomy, George Emil Palade University of Medicine, Pharmacy, Science, and Technology of Targu Mures, Targu Mures, Romania; 5 Doctoral School of Medicine and Pharmacy, George Emil Palade University of Medicine, Pharmacy, Science, and Technology of Targu Mures, Targu Mures, Romania; 6 Department of Physiology, George Emil Palade University of Medicine, Pharmacy, Science, and Technology of Targu Mures, Targu Mures, Romania; 7 Department of Pathophysiology, George Emil Palade University of Medicine, Pharmacy, Science, and Technology of Targu Mures, Targu Mures, Romania

**Keywords:** abdominal aortic aneurysm, biomarkers, CONUT score, nutritional status, open repair, prognostic nutritional index, vascular surgery

## Abstract

**Introduction:**

Abdominal aortic aneurysms (AAA) are a potentially fatal vascular condition defined by an aortic diameter exceeding 3.0 cm or demonstrating a ≥50% increase in the normal diameter. This study aims to evaluate the impact of preoperative nutritional status on rupture at presentation and early postoperative complications.

**Methods:**

This retrospective, monocentric, observational study included 125 AAA patients admitted for open surgical repair (OSR). Demographic data, comorbidities, and risk factors were obtained from the hospital’s electronic database. Nutritional status was quantified using albumin, total protein, Prognostic Nutritional Index (PNI), and CONUT Score. Primary outcomes were postoperative AKI and 30-day mortality.

**Results:**

The average age was 72.6 ± 7.5 years, with 83.2% of patients being male. Rupture at presentation occurred in 56.0%, AKI in 20.0%, and the 30-day mortality rate was 47.2%. Patients with poor outcomes had lower levels of albumin, total protein, and PNI, and higher CONUT Score (all *p* < 0.05). ROC curve analysis showed that albumin, total protein, and PNI had strong correlations with rupture and 30-day mortality, with AUC values of approximately 0.82–0.98, whereas CONUT Score demonstrated a moderate association. In multivariate analyses, lower albumin, serum total protein, and PNI remained independently associated with rupture and 30-day mortality. Meanwhile, a higher CONUT Score remained independently associated with rupture but not with 30-day mortality after full adjustment.

**Conclusion:**

Poor preoperative nutritional status is strongly associated with aneurysm rupture at presentation and increased 30-day mortality after OSR. However, in ruptured cases, these biomarkers may reflect acute hemorrhage and physiological stress rather than baseline nutritional status alone.

## 1 Introduction

Abdominal aortic aneurysms (AAA) are a prevalent and potentially fatal vascular condition defined by progressive, irreversible dilation of the abdominal aorta (1–3). Conventionally, an AAA is diagnosed when the aortic diameter exceeds 3.0 cm or demonstrates a ≥ 50% increase relative to the expected normal diameter adjusted for age, sex, and body size. The majority of AAA are located in the infrarenal aorta, reflecting regional variations in vascular wall structure and biomechanical stress. Most aneurysms develop gradually and remain asymptomatic for extended periods, often identified incidentally on imaging or through organized screening programs (2, 4, 5). While chronic expansion is the usual disease course, rupture is the most severe and potentially fatal complication. Ruptured abdominal aortic aneurysms (rAAA) are medical emergencies with high prehospital death rates (4–6). Even patients who survive long enough to receive definitive treatment often have poor outcomes, highlighting the key difference between elective and emergency AAA management (7). Correctly classifying and promptly detecting high-risk aneurysms is crucial for enhancing patient survival.

The diameter of the aneurysm remains the most reliable predictor of rupture, with elective repair typically recommended at thresholds of ≥5.5 cm in males and ≥5.0 cm in females (8, 9). AAA mainly affects older adults, with a significant male predominance (2, 10, 11). The prevalence reaches nearly 8% in men over 65, although women tend to experience ruptures at smaller diameters and face worse outcomes (12, 13). Although incidence and mortality have decreased in many high-income countries–mainly due to reduced smoking and better screening–rAAA still results in a considerable number of deaths worldwide (14–16). It is estimated that up to 50% of patients with rupture die before reaching the hospital, and perioperative mortality among treated patients remains high, even in specialized centers (4, 17, 18).

The European Society for Vascular Surgery (ESVS) recommends an individualized approach to AAA management, balancing aneurysm characteristics, patient comorbidities, life expectancy, and anatomical suitability when selecting between open surgical repair (OSR) and endovascular aneurysm repair (EVAR) (19, 20). EVAR is favored in patients with suitable anatomy due to lower short-term morbidity and mortality, whereas OSR remains essential for younger, low-risk patients, those with unfavorable anatomy, or aneurysms associated with infection or connective tissue disorders. For complex aneurysms, advanced techniques such as fenestrated or branched EVAR are recommended in experienced, high-volume centers (5, 17, 19, 21).

Recent studies have increasingly acknowledged sarcopenia, characterized by decreased skeletal muscle mass and function, as a marker of frailty and diminished physiological reserve in patients undergoing AAA repair. Recent evidence indicates that sarcopenia is independently correlated with higher perioperative morbidity, extended hospitalization, and decreased short- and long-term survival following both OSR and EVAR (22–26). Furthermore, hypoalbuminemia, a widely used marker of malnutrition, has been associated with increased perioperative morbidity, longer hospital stay, and elevated mortality following both open and endovascular AAA repair (27). Moreover, low Prognostic Nutritional Index (PNI), reflecting impaired nutritional and immunological status, has also been linked to poorer postoperative and long-term outcomes in surgical cohorts (28, 29).

This study aims to evaluate the impact of preoperative nutritional status on adverse outcomes in patients undergoing open AAA repair, with particular emphasis on rupture at presentation and early postoperative complications. By analyzing routinely available nutritional biomarkers and validated nutritional scores, including serum albumin, serum total protein, PNI, and CONUT score, the study investigates their associations with acute kidney injury and 30-day mortality. Additionally, a secondary analysis will be performed to identify clinical and laboratory factors associated with perioperative adverse events in patients with abdominal aortic aneurysm.

## 2 Methods

### 2.1 Study design

This retrospective, monocentric, observational study included all patients diagnosed with AAA who were admitted to the Vascular Surgery Department at the Emergency Clinical County Hospital of Târgu Mureș for OSR from January 2019 to December 2024. Demographic data, including age and sex, as well as comorbidities and common risk factors, were extracted from the hospital’s electronic database. The maximum AAA diameter was measured via preoperative computed tomography angiography (CTA). Laboratory data included in the current analysis were obtained within 12 h of surgery. For patients presenting to the emergency department with rAAA, laboratory analyses conducted at presentation were recorded. During the study period, 198 patients with AAA were admitted to the Vascular Surgery Department. Of these, 49 patients underwent EVAR and were excluded, and 24 patients were excluded due to incomplete laboratory or clinical data. The final study cohort consisted of 125 patients who underwent open surgical repair and had complete baseline data available for analysis.

### 2.2 Data collection

Demographic characteristics, anthropometric measures – body mass index (BMI), medical history, and laboratory parameters were systematically retrieved from the hospital’s electronic medical records. Cardiovascular comorbidities assessed included hypertension, atrial fibrillation, ischemic heart disease, chronic heart failure, valvular heart disease, history of myocardial infarction, history of stroke, and peripheral arterial disease. In addition, relevant non-cardiovascular comorbid conditions such as chronic kidney disease, active or previous malignancy, chronic obstructive pulmonary disease, and diabetes mellitus were recorded. Established risk factors, including obesity and current smoking status, were also documented.

Preoperative laboratory evaluations comprised white blood cell count (WBC), alanine aminotransferase (ALT), aspartate aminotransferase (AST), serum electrolytes (potassium and sodium), glucose, total cholesterol, blood urea nitrogen (BUN), creatinine, hemoglobin, hematocrit, differential leukocyte counts (neutrophils, lymphocytes, monocytes), and platelet count (PLT). Laboratory data were obtained at emergency presentation for rAAA patients and within 12 h prior to surgery for elective/unruptured cases. Additionally, data on the number of red blood cell (RBC) transfusions per patient were extracted from the hospital’s electronic database, and the length of stay was documented.

### 2.3 Nutritional biomarkers

Baseline nutritional status was assessed using routinely available clinical laboratory parameters. Serum albumin and total serum protein levels were measured as indicators of protein status and systemic nutritional reserve. Additionally, the PNI, the Controlling Nutritional Status (CONUT) score, and the neutrophil-to-lymphocyte ratio (NLR) were calculated using established, validated formulas reported in the literature (30–33), with the specific formulas for the biomarkers presented in Supplementary Table S1. These indices combine biochemical and immune-related variables, enabling the identification of patients at risk of malnutrition. The integration of these markers facilitated a clinically meaningful stratification of nutritional status at the time of evaluation.

### 2.4 Outcome

The primary outcomes of the study comprised rupture of the AAA at admission and major adverse events of direct surgical relevance, including the development of postoperative acute kidney injury (AKI), and 30-day all-cause mortality following OSR. Postoperative AKI was defined as stage 1 or higher according to the Kidney Disease: Improving Global Outcomes (KDIGO) criteria (34). These outcomes were selected to capture both the severity of disease at admission and early postoperative morbidity and mortality associated with surgical management. All adverse events were systematically recorded throughout the 30-day follow-up period.

### 2.5 Statistical analysis

Statistical analyses were performed using SPSS for Mac OS (version 29.0.2.0; SPSS Inc., Chicago, IL, United States). Continuous variables, including age, length of hospital stay, and maximum AAA diameter, are presented as mean ± standard deviation (SD). Laboratory parameters are reported as median values with interquartile ranges (Q1–Q3). Differences between continuous variables were assessed using either the Student’s *t*-test or the Mann–Whitney *U*-test, as appropriate. Categorical variables were compared using the chi-square test. Receiver operating characteristic (ROC) curve analysis was conducted to investigate the association between preoperative nutritional biomarkers and major adverse events following open surgical repair (OSR) of AAA. Optimal cut-off values for the baseline nutritional and inflammatory biomarkers at ROC curve analyses were determined using the Youden Index, defined as the point maximizing the sum of sensitivity and specificity (Youden Index = sensitivity + specificity − 1). Univariate analyses were performed to evaluate the association between variables of interest and rAAA and 30-day mortality. Multivariate analyses were performed using hierarchical adjustment models with progressively included covariates. Model 1 included age and sex, while Model 2 additionally included cardiovascular risk factors (atrial fibrillation, history of myocardial infarction, chronic kidney disease, diabetes mellitus, and active smoking). Model 3 further included laboratory and perioperative variables (creatinine, hemoglobin, NLR, and red blood cell transfusion), and Model 4 additionally incorporated rupture status for the analysis of 30-day mortality. Because serum albumin, serum total protein, PNI, and CONUT Score are physiologically related, these biomarkers were analyzed in separate regression models to reduce the risk of multicollinearity and to improve model stability. The multivariable analyses were designed to explore independent associations while limiting excessive model complexity. All statistical tests were two-tailed, and a *p*-value < 0.05 was considered statistically significant.

## 3 Results

The study included 125 patients with an average age of 72.6 ± 7.53 years. Among them, 104 (83.2%) were male and 21 (16.8%) were female, with a mean BMI value of 29.36 ± 7.69. Preoperative CTA revealed a mean maximal abdominal aortic aneurysm (AAA) diameter of 9.25 ± 4.37 cm. Hypertension was the most common cardiovascular comorbidity, affecting 98 patients (78.4%), followed by ischemic heart disease in 65 patients (44.8%) and chronic heart failure in 38 patients (30.4%). Other prevalent comorbidities included diabetes mellitus in 53 patients (42.4%), chronic obstructive pulmonary disease and malignancy each in 18 patients (14.4%), and chronic kidney disease in 17 patients (13.6%) (Table 1). At admission, 70 patients (56.0%) arrived at the emergency department with rAAA. After OSR, AKI developed in 25 patients (20.0%). The average length of hospital stay was 8.78 ± 7.83 days (Table 1).

**Table 1:** All the characteristics of the patients enrolled in the study presented based on 30-days mortality.

Variables	All patients *n* = 125	30-days outcome		*p*-value
		Survival *n* = 66	Mortality *n* = 59	
Age, mean ± SD	72.6 ± 7.53	70.98 ± 6.75	74.41 ± 7.99	**0.011**
Male, *n* (%)	104 (83.20%)	56 (84.85%)	48 (81.36%)	0.602
Female, n (%)	21 (16.80%)	10 (15.15%)	11 (18.64%)	
AAA diameter, mean ± SD	9.25 ± 4.37	9.38 ± 2.44	9.13 ± 2.64	0.847
Weight (kg)*, mean ± SD	86.91 ± 24.77	83.51 ± 20.67	89.51 ± 27.45	0.248
Height (cm)*, mean ± SD	171.59 ± 8.13	173.11 ± 7.49	170.43 ± 8.47	0.117
BMI*, mean ± SD	29.36 ± 7.69	27.76 ± 6.25	29.36 ± 7.69	0.087
Comorbidities and risk factors, *n* (%)				
Hypertension	98 (78.40%)	53 (80.30%)	45 (76.27%)	0.584
Atrial fibrillation	18 (14.40%)	4 (6.06%)	14 (23.73%)	**0.005**
Ischemic heart disease	56 (44.80%)	32 (48.48%)	24 (40.68%)	0.381
Chronic heart failure	38 (30.40%)	19 (28.79%)	19 (32.20%)	0.679
Valvulopathy	26 (20.80%)	18 (27.27%)	8 (13.56%)	0.059
History of myocardial infarction	21 (16.8%)	6 (9.09%)	15 (25.42%)	**0.015**
Chronic kidney disease	18 (14.40%)	5 (7.58%)	13 (22.03%)	**0.022**
Chronic obstructive pulmonary disease	17 (13.60%)	8 (12.12%)	9 (15.25%)	0.610
History of stroke	12 (9.60%)	4 (6.06%)	8 (13.56%)	0.155
Diabetes mellitus	53 (42.40%)	22 (33.33%)	31 (52.54%)	**0.030**
Malignancy	18 (14.40%)	11 (16.67%)	7 (11.86%)	0.455
Peripheral arterial disease	32 (25.60%)	19 (28.79%)	13 (22.03%)	0.388
Active smoking	44 (35.20%)	23 (34.84%)	21 (35.59%)	0.931
Obesity	20 (16.00%)	9 (13.64%)	11 (18.64%)	0.446
Laboratory data, median (Q1-Q3)				
WBC	10.71 (8.43–14.23)	8.94 (7.75–10.9)	13.16 (10.93–17.15)	**<0.001**
ALT	17.5 (11.85–28.5)	17.0 (12.0–24.0)	18.50 (11.6–42.75)	0.202
AST	22.0 (16.2–35.0)	20.0 (17.0–27.2)	29.0 (15.75–64.0)	**0.024**
Potassium mmol/l	4.44 (4.0–4.85)	4.30 (4.01–4.61)	4.77 (3.98–5.25)	**0.014**
Sodium mmol/l	140.1 (137.3–142.0)	140.0 (137.15–141.9)	141.0 (138.0–144.0)	0.219
Glucose (mg/dL)	134.0 (104.0–184.0)	113.0 (102.0–142.0)	164.0 (132.0–230.5)	**<0.001**
Total cholesterol (mg/dL)	142.9 (118.2–185.8)	154.0 (120.8–190.2)	123.0 (95.7–143.5)	0.262
BUN (mg/dL)	43.35 (34.21–59.44)	38.90 (29.96–51.7)	51.36 (38.52–70.62)	**<0.001**
Creatinine (mg/dL)	1.15 (0.88–1.85)	1.04 (0.80–1.26)	1.63 (1.02–2.22)	**<0.001**
Hemoglobin g/dL	11.95 (8.88–13.62)	12.82 (11.2–14.4)	9.53 (7.84–11.79)	**<0.001**
Hematocrit %	35.0 (27.25–40.72)	38.8 (33.82–43.45)	28.8 (24.63–35.0)	**<0.001**
Neutrophils × 10^3^/uL	7.71 (5.50–11.91)	6.36 (5.06–8.85)	10.66 (6.72–13.97)	**<0.001**
Lymphocytes × 10^3^/uL	1.60 (1.08–2.31)	1.69 (1.18–2.39)	1.52 (1.01–2.06)	0.594
Monocyte × 10^3^/uL	0.69 (0.53–0.99)	0.67 (0.54–0.77)	0.78 (0.52–1.11)	0.101
PLT × 10^3^/uL	194.0 (153.5–244.25)	213.0 (168.5–256.6)	173.95 (136.3–230.0)	**0.008**
NLR	5.12 (2.68–9.88)	3.36 (2.26–6.23)	7.03 (3.84–12.36)	**0.002**
Treatment and outcome, *n* (%)				
rAAA	70 (56.00%)	17 (25.76%)	53 (89.83%)	**<0.001**
Post-operative AKI	25 (20.00%)	8 (12.12%)	17 (28.81%)	**0.020**
RBC transfusion	3.57 ± 2.89	2.74 ± 2.67	4.49 ± 3.96	**<0.001**
Length of stay, mean ± SD	8.78 ± 7.83	12.52 ± 9.33	5.58 ± 4.23	**<0.001**

* Value of weight, height, and BMI is available only for a group of 83 patients from the entire cohort. Bold values indicate statistically significant results (*p* < 0.05).

At 30 days following OSR for AAA, 59 patients experienced a poor outcome, defined by 30-day mortality. Patients in this group were significantly older compared with survivors (*p* = 0.011), while no difference was observed in sex distribution between groups (*p* = 0.602). With regard to comorbidities, patients who died within 30 days had a significantly higher prevalence of atrial fibrillation (*p* = 0.005), a prior history of myocardial infarction (*p* = 0.015), and diabetes mellitus (*p* = 0.030). No statistically significant differences were identified for other comorbid conditions or cardiovascular risk factors (Table 1). Analysis of pre-operative laboratory parameters revealed that the poor outcome group presented significantly higher values of white blood cell count (*p* < 0.001), aspartate aminotransferase (*p* = 0.024), serum potassium (*p* = 0.014), glucose (*p* < 0.001), BUN (*p* < 0.001), creatinine (*p* < 0.001), and neutrophil count (*p* < 0.001). Conversely, significantly lower levels of hemoglobin (*p* < 0.001), hematocrit (*p* < 0.001), and platelet count (*p* = 0.008) were observed in patients with 30-day mortality (Table 1). Regarding systemic inflammation status, patients with poor 30-day outcomes have a higher NLR (*p* = 0.002) (Table 1). As expected, ruptured AAA was significantly more frequent among patients with poor outcomes (*p* < 0.001). In addition, the incidence of postoperative acute kidney injury was significantly higher in this group compared with survivors (*p* = 0.020) (Table 1).

**Figure 1:**
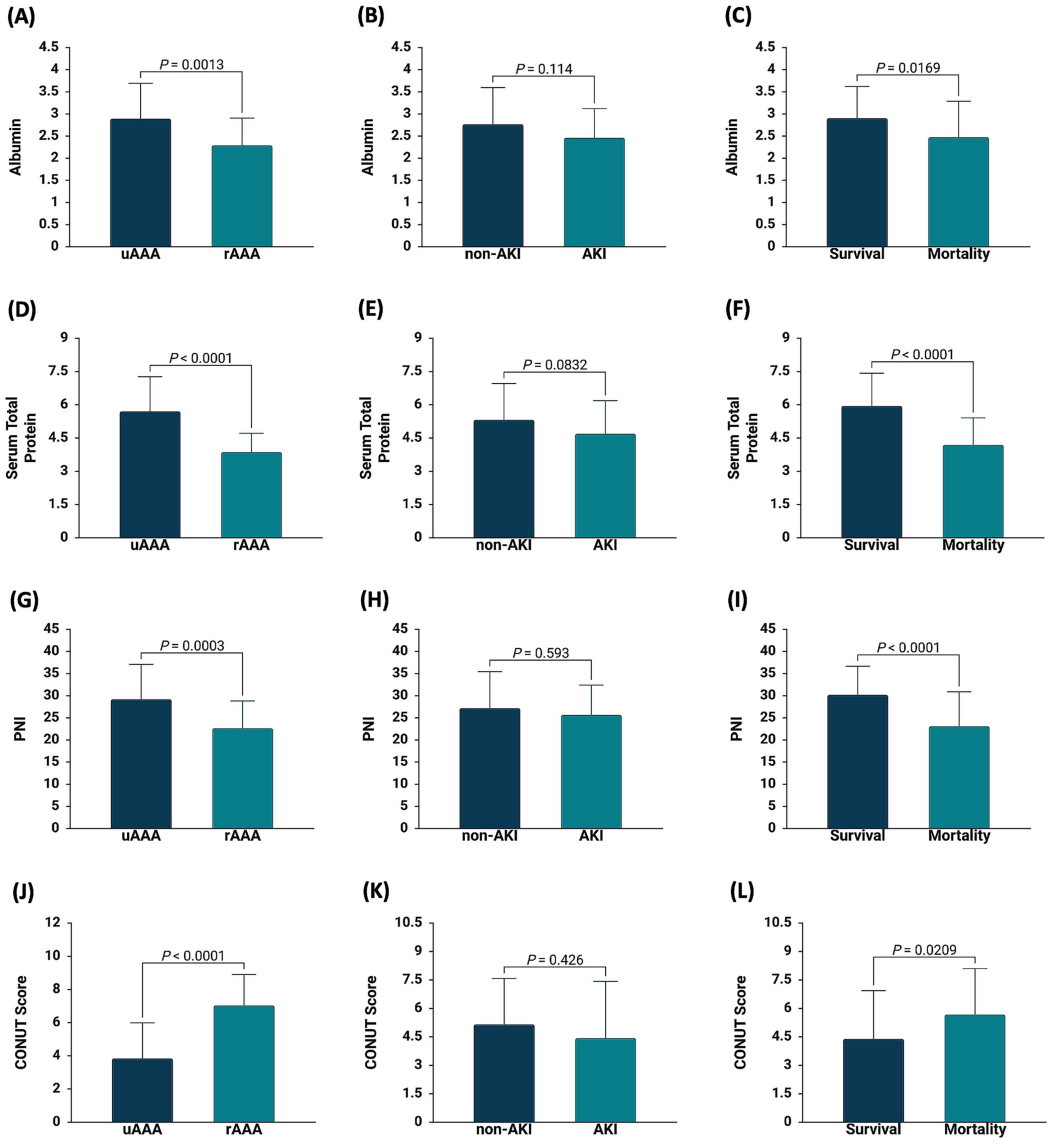
Association of nutritional biomarkers at baseline with aneurysm status, postoperative acute kidney injury, and 30-day mortality following OSR for AAA. Bar plots illustrate preoperative serum albumin **(A–C)**, serum total protein **(D–F)**, prognostic nutritional index (PNI) **(G–I)**, and CONUT score **(J–L)** stratified by aneurysm presentation (uAAA vs. rAAA), postoperative acute kidney injury (non-AKI vs. AKI), and 30-day outcome (survival vs. mortality). Data are presented as mean ± standard deviation. *p*-values indicate between-group comparisons.

Patients presenting with rAAA exhibited significantly lower serum albumin (*p* = 0.0013) (Figure 1A), serum total protein (*p* < 0.0001) (Figure 1D), and PNI (*p* = 0.0003) (Figure 1G), alongside significantly higher CONUT Score (*p* < 0.0001) (Figure 1J), compared with patients with unruptured AAA (uAAA). In contrast, no statistically significant differences were observed in albumin (Figure 1B), serum total protein (Figure 1E), PNI (Figure 1H), or CONUT Score (Figure 1K) between patients who developed postoperative AKI and those who did not. Importantly, patients who died within 30 days after OSR had significantly lower serum albumin (*p* = 0.0169) (Figure 1C), serum total protein (*p* < 0.0001) (Figure 1F), and PNI (*p* < 0.0001) (Figure 1I), together with significantly higher CONUT Score (*p* = 0.0209) (Figure 1L), compared with survivors. These results highlight a strong association between poor preoperative nutritional status and increased short-term mortality.

**Figure 2:**
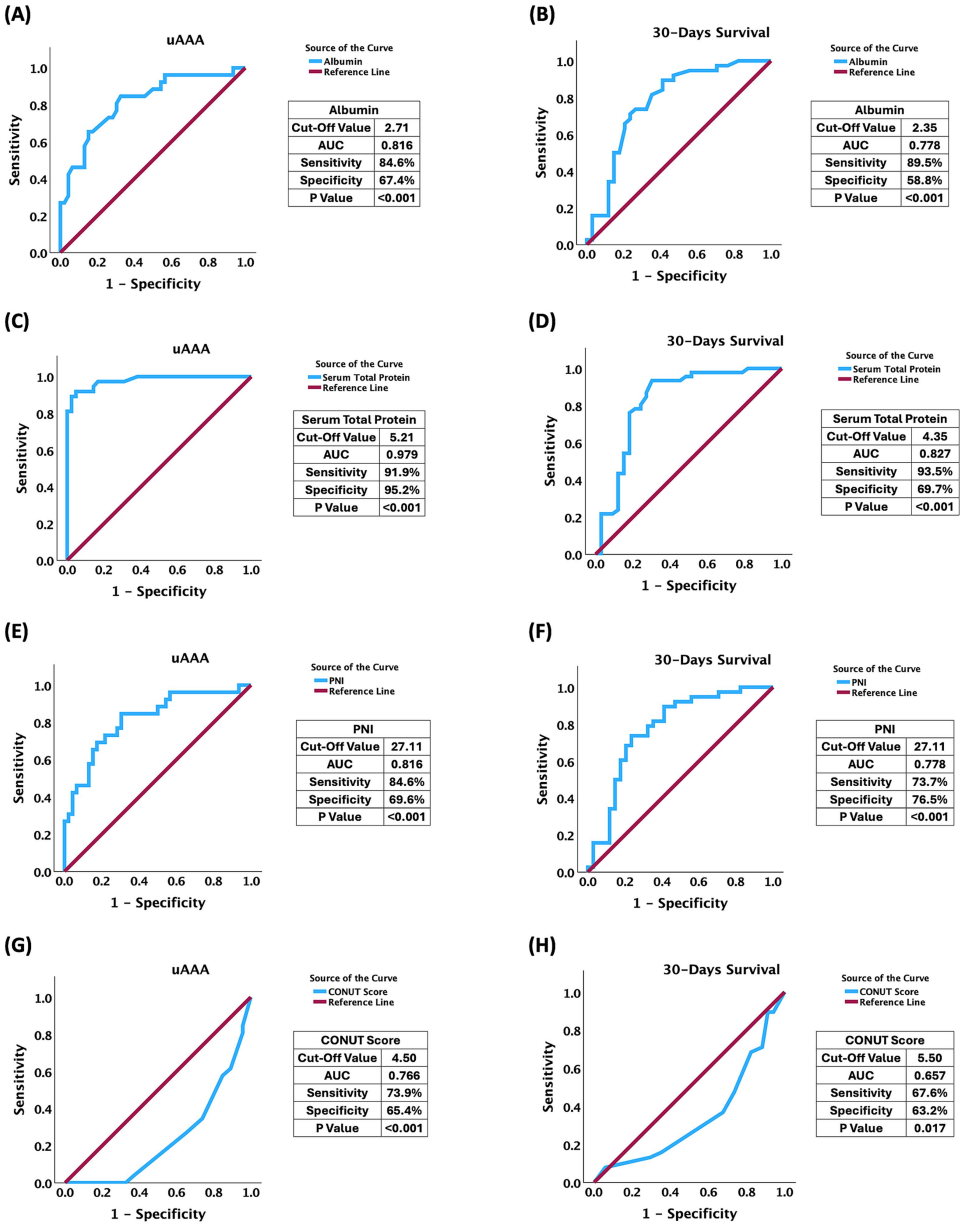
Receiver operating characteristic (ROC) curve analysis of preoperative nutritional biomarkers for aneurysm status at baseline and 30-day survival following OSR for AAA. ROC curves illustrate the difference of preoperative serum albumin **(A,B)**, serum total protein **(C,D)**, PNI **(E,F)**, and CONUT score **(G,H)** for uAAA (left panels) and 30-day survival (right panels). Area under the curve (AUC), optimal cut-off values, sensitivity, specificity, and corresponding *p*-values are reported for each biomarker. The diagonal line represents the reference line.

At ROC Curve analysis, albumin, serum total protein, and PNI exhibited good discriminative ability for differentiating unruptured from ruptured AAA, with AUC values ranging from 0.816 to 0.979 (for all *p* < 0.05) (Figures 2A,C,E), indicating strong associations between impaired nutritional status and aneurysm rupture at baseline. The CONUT Score demonstrated a moderate association with rupture status (AUC 0.766, *p* < 0.001) (Figure 2G). Similarly, all evaluated biomarkers were significantly associated with 30-day survival following OSR. Lower albumin, serum total protein, and PNI values, as well as higher CONUT Score, were associated with increased short-term mortality, with AUCs indicating good prognostic accuracy (for all *p* < 0.05) (Figures 2B,D,F,H). In contrast, the CONUT Score demonstrated modest but statistically significant prognostic value. Overall, these findings support the clinical utility of preoperative nutritional and inflammation-related indices—particularly serum total protein, albumin, and PNI—as effective tools for risk stratification of aneurysm rupture and early postoperative mortality after AAA repair. The CONUT Score, although less robust, may still provide complementary prognostic information within a comprehensive preoperative assessment. Detailed ROC curve metrics, including AUCs, 95% CI AUC, optimal cutoff values, sensitivity, and specificity, are provided in Supplementary Table S2.

Spearman correlation analysis demonstrated significant interrelationships among baseline inflammatory, nutritional, hematological, and renal parameters, highlighting the integrated nature of these physiological domains (Supplementary Table S3). Regarding anthropometric markers, BMI showed moderate positive correlations with serum albumin (*r* = 0.358, *p* = 0.012) and PNI (*r* = 0.351, *p* = 0.014), suggesting that higher BMI values are associated with a more favorable nutritional status. Baseline hemoglobin levels exhibited strong positive correlations with albumin (*r* = 0.485, *p* < 0.001), serum total protein (*r* = 0.657, *p* < 0.001), and PNI (*r* = 0.488, *p* < 0.001). In contrast, hemoglobin was negatively correlated with creatinine (*r* = −0.323, *p* < 0.001), NLR (*r* = −0.398, *p* < 0.001), and CONUT Score (*r* = −0.422, *p* < 0.001). These findings suggest that anemia is not merely a hematologic abnormality but may reflect impaired nutritional status, systemic inflammation, and reduced renal function. Preoperative anemia may therefore represent a surrogate marker of diminished physiological reserve. In patients with AAA, particularly those presenting with rAAA, this may indicate increased susceptibility to perioperative stress, renal injury, and adverse clinical outcomes rather than isolated erythropoietic dysfunction. Renal function at baseline was positively associated with systemic inflammation, as indicated by the correlation with NLR (*r* = 0.263, *p* = 0.003), and inversely associated with serum total protein (*r* = −0.398, *p* < 0.001). These relationships support the concept that renal impairment may coexist with inflammatory activation and disrupted protein homeostasis. Serum albumin emerged as a central integrative biomarker, reflecting the interplay between nutritional and inflammatory pathways. Its negative correlation with NLR (*r* = −0.287, *p* = 0.015) reinforces its role as a negative acute-phase reactant influenced by inflammatory signaling. Serum total protein showed similar correlation patterns, underscoring its association with overall protein balance and its effects on inflammation. Notably, NLR appeared as a dominant variable linking inflammation with both nutritional and hematological deterioration. Higher baseline inflammatory burden correlated with poorer nutritional indices, as evidenced by its negative association with PNI (*r* = −0.300, *p* = 0.011) and strong positive association with CONUT Score (*r* = 0.539, *p* < 0.001).

In univariate logistic regression analysis, increasing age was significantly associated with both aneurysm rupture (OR: 1.55, *p* = 0.021) and 30-day mortality (OR: 1.61, *p* = 0.013), indicating that older patients were at higher risk for both adverse presentations and outcomes. In contrast, sex was not significantly associated with either rAAA or short-term mortality. Among comorbidities, atrial fibrillation emerged as a strong predictor of both rAAA (OR: 7.85, *p* = 0.008) and 30-day mortality (OR: 4.82, *p* = 0.009). Similarly, a history of myocardial infarction was significantly associated with increased odds of aneurysm rupture (OR: 2.96, *p* = 0.048) and mortality within 30 days (OR: 3.41, *p* = 0.019). Chronic kidney disease was not associated with rAAA but was significantly linked to 30-day mortality (OR: 3.44, *p* = 0.027), underscoring its prognostic relevance in the postoperative period. In contrast, chronic obstructive pulmonary disease showed no significant association with either outcome (Table 2). Preoperative hematological parameters demonstrated strong protective associations. Higher hemoglobin and hematocrit levels were independently associated with significantly lower odds of both rAAA and 30-day mortality. Specifically, each 1 SD increase in hemoglobin was associated with a marked reduction in the risk of rupture (OR: 0.23, *p* < 0.001) and mortality (OR: 0.21, *p* < 0.001). Similarly, higher hematocrit levels were associated with reduced odds of rAAA (OR: 0.14, *p* < 0.001) and 30-day mortality (OR: 0.34, *p* < 0.001) (Table 2).

**Table 2:** Univariate analysis: the association of demographic data, comorbidities, laboratory data, and adverse events following OSR in AAA patients.

Variables	rAAA			30-day mortality		
	OR	95% CI	*p*-value	OR	95% CI	*p*-value
Age*	1.55	1.07–2.24	**0.021**	1.61	1.11–2.34	**0.013**
Male	0.58	0.22–1.56	0.284	0.78	0.31–1.99	0.603
Atrial fibrillation	7.85	1.72–35.83	**0.008**	4.82	1.48–15.62	**0.009**
History of myocardial infarction	2.96	1.01–8.68	**0.048**	3.41	1.22–9.48	**0.019**
Chronic kidney disease	1.69	0.59–4.83	0.328	3.44	1.15–10.36	**0.027**
Chronic obstructive pulmonary disease	0.87	0.31–2.42	0.785	1.31	0.47–3.64	0.611
Hemoglobin*	0.23	0.12-0.44	**<0.001**	0.21	0.11–0.39	**<0.001**
Hematocrit*	0.14	0.08-0.28	**<0.001**	0.34	0.22–0.54	**<0.001**

* expressed per 1 SD increase in baseline. Bold values indicate statistically significant results (*p* < 0.05).

Elevated baseline levels of albumin (OR: 0.21, *p* < 0.001 and OR: 0.32, *p* < 0.001), serum total protein (OR: 0.01, *p* < 0.001 and OR: 0.25, *p* < 0.001), and PNI (OR: 0.21, *p* < 0.001 and OR: 0.32, *p* < 0.001) were associated with a lower risk of rAAA and 30-day mortality (Table 3). Furthermore, multivariate analysis confirmed that these associations remain significant after comprehensive adjustment for potential confounders, indicating that maintained nutritional and immunologic reserves provide considerable protection independent of demographic variables and cardiovascular comorbidities. Conversely, higher CONUT Score, which indicate greater malnutrition severity, were associated with higher risk of rAAA (OR: 2.91, *p* < 0.001) and 30-day mortality (OR: 1.72, *p* = 0.037). Moreover, a higher CONUT Score was associated with rAAA after fully adjusted models (OR: 2.48, *p* = 0.014), but not with 30-day mortality risk (OR:1.37, *p* = 0.320) (Table 3). In Model 3, after adjustment for demographic variables, cardiovascular risk factors, baseline renal function, hemoglobin level, NLR, and transfusion requirement, serum albumin (OR: 0.27, *p* = 0.009 and OR: 0.19, *p* = 0.006) and PNI (OR: 0.27, *p* = 0.009 and OR: 0.19, *p* = 0.006) remained significantly associated with both rAAA and 30-day mortality, indicating that these biomarkers retain prognostic relevance even after accounting for major clinical confounders. In contrast, the CONUT Score was no longer significantly associated with outcomes, while serum total protein showed only a borderline association with mortality. In Model 4, which additionally incorporated aneurysm rupture status, albumin (OR: 0.18, *p* = 0.008) and PNI (OR: 0.18, *p* = 0.008) remained associated with 30-day mortality, whereas serum total protein (OR: 0.75, *p* = 0.661) and CONUT Score (OR: 2.01, *p* = 0.180) did not, suggesting that albumin-based indices may provide more robust prognostic information beyond the dominant effect of rupture presentation.

**Table 3:** Multivariate analysis: the association between baseline nutritional biomarkers and poor outcomes following AAA open surgery in all patients.

Variables		rAAA			30-day mortality		
		OR*	95% CI	*p*-value	OR*	95% CI	*p*-value
Albumin	Unadjusted	0.21	0.10–0.48	**<0.001**	0.32	0.17–0.61	**<0.001**
	Model 1	0.19	0.09–0.46	**<0.001**	0.31	0.15–0.59	**<0.001**
	Model 2	0.23	0.11–0.55	**0.001**	0.28	0.13–0.63	**0.002**
	Model 3	0.27	0.10–0.73	**0.009**	0.19	0.06–0.062	**0.006**
	Model 4	-	-	-	0.18	0.05–0.63	**0.008**
Serum total protein	Unadjusted	0.01	0.01–0.08	**<0.001**	0.25	0.13–0.48	**<0.001**
	Model 1	0.02	0.01–0.11	**<0.001**	0.27	0.15–0.52	**<0.001**
	Model 2	0.04	0.02–0.14	**0.006**	0.35	0.17–0.68	**0.002**
	Model 3	0.02	0.01–0.045	**0.007**	0.44	0.18–1.09	0.075
	Model 4	-	-	-	0.75	0.21–2.69	0.661
PNI	Unadjusted	0.21	0.10–0.47	**<0.001**	0.32	0.17–0.60	**<0.001**
	Model 1	0.19	0.10–0.45	**<0.001**	0.31	0.15–0.58	**<0.001**
	Model 2	0.22	0.09–0.54	**0.001**	0.27	0.13–0.62	**0.002**
	Model 3	0.27	0.11–0.73	**0.009**	0.19	0.06–0.62	**0.006**
	Model 4	-	-	-	0.18	0.05–0.64	**0.008**
CONUT score	Unadjusted	2.91	1.58–5.32	**<0.001**	1.72	1.03–2.84	**0.037**
	Model 1	2.83	1.51–5.29	**0.001**	1.57	0.92–2.69	0.096
	Model 2	2.48	1.21–5.13	**0.014**	1.37	0.74–2.53	0.320
	Model 3	1.71	0.69–4.20	0.242	1.95	0.74–5.09	0.174
	Model 4	-	-	**-**	2.01	0.73–5.55	0.180

Model 1, age and sex; Model 2, age, sex, and CV risk factors (atrial fibrillation, history of myocardial infarction, CKD, diabetes, and active smoking); Model 3, age, sex, and CV risk factors (atrial fibrillation, history of myocardial infarction, CKD, diabetes, and active smoking), creatinine, hemoglobin, NLR, and RBC transfusion. Model 4, age, sex, and CV risk factors (atrial fibrillation, history of myocardial infarction, CKD, diabetes, and active smoking), creatinine, hemoglobin, NLR, RBC transfusion, and rAAA. * expressed per 1 SD increase in baseline. Bold values indicate statistically significant results (*p* < 0.05).

## 4 Discussion

The primary findings of this study indicate that preoperative malnutrition is a significant and independent factor associated with aneurysm instability and 30-day postoperative mortality. These results support the incorporation of nutritional biomarkers into perioperative risk assessment for patients undergoing OSR of AAA. Higher baseline levels of albumin, serum total protein, and PNI were repeatedly associated with significantly lower odds of ruptured AAA and 30-day mortality in both unadjusted and fully adjusted models. However, these biomarkers may reflect overall physiological reserve and acute stress response rather than nutritional status alone in patients presenting with rAAA. This suggests that better nutritional and immune reserves provide substantial protection, regardless of age, sex, and cardiovascular risk factors. Conversely, higher CONUT Score, which indicate greater degrees of malnutrition, were independently associated with increased odds of ruptured AAA. However, their association with 30-day mortality was not statistically significant after adjustment, implying partial confounding by comorbid disease burden. Collectively, these findings suggest that preoperative malnutrition is associated with rupture at presentation of an aneurysm and early postoperative mortality, highlighting the clinical importance of nutritional biomarkers in risk stratification and perioperative management of patients undergoing open AAA repair. It should also be noted that the present cohort represents a high-risk population, as more than half of the patients presented with rAAA, and the early mortality rate was high, which may influence the observed associations.

From a mechanistic perspective, sarcopenia reflects chronic inflammation, impaired metabolic reserve, and reduced cardiopulmonary and immunological resilience, all of which limit the ability to tolerate operative stress and recover from complications (27, 35–37). These effects are particularly relevant in elderly patients and those undergoing complex or urgent repair, including ruptured AAA. Preoperative nutritional status has also emerged as an important determinant of outcomes in patients undergoing AAA repair. Hypoalbuminemia, a widely used marker of malnutrition, has been associated with increased perioperative morbidity, longer hospital stay, and elevated mortality following both open and endovascular AAA repair (27). Low PNI, reflecting impaired nutritional and immunological status, has also been linked to poorer postoperative and long-term outcomes in surgical cohorts (28). Additionally, elevated CONUT Score have been independently associated with higher rates of major complications and reduced mid-term survival after AAA repair (29, 33). These indices integrate measures of protein reserves, lymphocyte counts, and cholesterol levels, thereby reflecting a combination of malnutrition, systemic inflammation, and impaired immune competence, factors that compromise physiological reserve and tolerance to surgical stress. Moreover, low albumin and PNI correlate with sarcopenia, diminished cardiopulmonary capacity, and reduced wound-healing potential, while higher CONUT Score may indicate ongoing catabolic states that exacerbate tissue vulnerability (38–40). Incorporating these nutritional and inflammatory biomarkers into preoperative assessment aligns with ESVS and SVS recommendations for individualized risk stratification, potentially identifying patients who may benefit from prehabilitation or nutritional optimization prior to elective or urgent AAA repair (7, 41). As such, albumin-based indices represent practical, readily available tools to refine prognostication and guide perioperative management in AAA populations.

Malnutrition is highly common among patients with vascular disease, and nutritional indices have consistently been linked to adverse outcomes after peripheral artery disease-related revascularization treatments (42–44). Recently, Morisaki et al. (45) and Itagaki et al. (46) published studies showing a positive link between malnutrition and 2-year major amputation rates and overall survival in patients with chronic threatening limb ischemia (CLTI). Additionally, Öcal et al. (47) found a significant connection between lower baseline PNI values and long-term all-cause mortality and major adverse events following carotid artery stenting (CAS). Further retrospective analyses (48) also revealed an association between lower PNI scores and the development of in-stent restenosis after CAS. Moreover, Çakmak et al. (49) reported that malnutrition was significantly associated with 30-day major adverse events after CAS in a cohort of 978 patients, using various nutritional and inflammatory markers, including PNI.

Our findings are consistent with accumulating evidence that objective malnutrition indices represent clinically meaningful risk markers in patients with AAA. Among the biomarkers most frequently evaluated in the literature, serum albumin has received particular attention (50–53). In a cohort of 678 patients, Park et al. (50) demonstrated that low serum albumin levels were associated with reduced one-year survival following elective OSR. Similarly, Yoon et al. (51) reported that postoperative hypoalbuminemia was linked to both postoperative AKI and mortality in patients with rAAA. In contrast to our observations, Xu et al. (53) identified serum albumin levels below 32 g/L as an independent risk factor for AKI in patients undergoing surgery for acute type A aortic dissection. While we did not detect a significant association between serum albumin levels and AKI, this discrepancy may be explained by differences in patient populations, as our cohort exclusively comprised individuals with AAA. Regarding the PNI, prior studies by Keskin et al. (54) and Lin et al. (55) demonstrated that lower baseline PNI values were associated with increased in-hospital mortality among patients with acute type A aortic dissection. In a dedicated rAAA cohort, Ye et al. (56) further showed that higher CONUT Score were independently associated with worse survival, reinforcing the concept that malnutrition, frailty, and inflammatory–catabolic burden contributes to adverse outcomes in emergency aneurysm surgery. In our study, CONUT exhibited a moderate association with aneurysm rupture but lost statistical significance for 30-day mortality after full adjustment. In contrast, albumin-based indices (serum albumin and PNI) remained robust predictors. These findings suggest that, in a predominantly emergency OSR population, acute-phase responses and hemorrhage-related physiological disturbances may attenuate the incremental prognostic contribution of cholesterol-derived components within the CONUT Score, whereas albumin and PNI may better capture short-term physiological reserve. No significant association was found between the evaluated nutritional biomarkers and postoperative AKI in the present study. This contrasts with previous reports in major vascular and aortic surgery, where hypoalbuminemia and poor nutritional status have been linked to higher rates of renal complications (51, 53). Several factors may explain this discrepancy. First, the relatively small sample size limits statistical power, particularly for subgroup analyses. Second, AKI following open AAA repair is multifactorial and may be primarily driven by intraoperative ischemia–reperfusion injury, hemodynamic instability, blood loss, and transfusion requirements rather than baseline nutritional reserve. Third, more than half of the cohort presented with rupture, a condition associated with severe hemorrhage, shock, and systemic inflammation, which may overshadow the influence of nutritional status on renal outcomes.

### 4.1 Study limitations

The current study has several notable limitations that should be acknowledged. Firstly, its retrospective nature, being conducted at a single center with a relatively small sample size, restricts the ability to generalize findings and may introduce selection bias. Secondly, only patients who underwent open surgical repair were included, with no comparison group of EVAR patients, and the short 30-day follow-up limits understanding of longer-term outcomes. Thirdly, residual confounding factors cannot be entirely ruled out; unmeasured variables such as inflammatory burden, frailty scores, or perioperative care might affect both nutritional status and patient outcomes. A further limitation of this study is the absence of consistently recorded perioperative timing and bleeding-related variables, including the interval from emergency department admission to surgical intervention, intraoperative blood loss, and detailed fluid resuscitation data. These factors are particularly relevant in ruptured AAA and may influence both laboratory parameters and clinical outcomes. Consequently, their omission may introduce residual confounding and should be considered when interpreting the observed associations. Lastly, nutritional status was evaluated through laboratory markers at one point in time, without considering body composition, functional measures of sarcopenia, or the effects of nutritional optimization strategies. Another important limitation is that laboratory values in rAAA patients were obtained at emergency department presentation, prior to any transfusion or perfusion-related intervention. Nonetheless, reverse causality cannot be excluded, as rupture and acute hemorrhage may independently alter albumin concentrations, protein levels, and immune cell distributions. Therefore, these biomarkers cannot be interpreted solely as indicators of chronic nutritional status, and the observed associations should be considered reflective of overall physiological reserve at presentation rather than predictors of rupture risk. Prospective studies incorporating outpatient pre-rupture laboratory data would be required to evaluate true rupture prediction. Moreover, the present cohort represents a high-risk population, with more than half of patients presenting with rAAA and a high early mortality rate. This reflects the case mix of a tertiary emergency referral center, where OSR is frequently performed in urgent situations; therefore, the results may not be generalizable to centers where EVAR predominates. Furthermore, although hierarchical multivariable models were used, the small sample size restricts the number of covariates that can be incorporated without risking overfitting. Therefore, these multivariable analyses should be considered exploratory, and their results need validation in larger, prospective cohorts.

## 5 Conclusion

In the present study, lower levels of albumin, total serum protein, and PNI, as well as higher CONUT scores, were associated with aneurysm rupture at admission and increased 30-day mortality, regardless of age, sex, and major cardiovascular comorbidities. These findings suggest that readily available nutritional biomarkers provide relevant prognostic information and may improve perioperative risk stratification beyond traditional clinical variables. Routine nutritional assessment during preoperative evaluation, along with strategies to optimize nutrition or prehabilitate, may represent important opportunities to improve outcomes in this high-risk surgical population. However, in patients presenting with ruptured AAA, these biomarkers may reflect reduced physiological reserve and acute stress response rather than nutritional status alone and should therefore be interpreted with caution until validated in prospective studies. Further prospective investigations with external validation are necessary before these markers can be reliably incorporated into clinical risk stratification or decision-making.

## Data Availability

The original contributions presented in the study are included in the article/Supplementary material, further inquiries can be directed to the corresponding author.
