# Supplementary material for: Preoperative nutritional status and its association with adverse events following open abdominal aortic aneurysm repair
Source: Front Nutr. 2026 Apr 16;13:1776781. doi: 10.3389/fnut.2026.1776781 (PMC13128428; doi:10.3389/fnut.2026.1776781)
Supplement: Supplementary file 1 [file Data_Sheet_1.pdf]

frontiers

Supplementary Material

1 Supplementary Data

2 Supplementary Figures and Tables

2.1 Supplementary Tables

Supplementary Table 1. Formulas and component variables for the PNI, NLR, and CONUT Score.

**Index**	**Formula**		**Component Variables/Units**	
**PNI**	10 x Serum Albumin + 0.005 x Total Lymphocyte Count		Serum Albumin (g/dL)<br>Total Lymphocyte Count ($\times 10^3$/μL)	
**NLR**	Total Neutrophil Count / Total Lymphocyte Count		Total Neutrophil Count ($\times 10^3$/μL)<br>Total Lymphocyte Count ($\times 10^3$/μL)	
**CONUT Score**				
	Normal	Mild	Moderate	Severe
**Serum Albumin (g/dL)**	≥3.50	3.00-3.49	2.50-2.99	<2.50
Score	0	2	4	6
**Total Lymphocyte Count ($\times 10^3$/μL)**	≥1.60	1.20-1.59	0.80-1.19	<0.80
Score	0	1	2	3
**Total Cholesterol (md/dL)**	≥180	140-179	100-139	<100
Score	0	1	2	3
Total Score	0-1	2-4	5-8	9-12

Supplementary Table 2. Receiver operating characteristic (ROC) curve analysis of nutritional biomarkers for study outcomes.

Biomarker	Outcomes	Cut-Off Value	Sensitivity (%)	Specificity (%)	AUC	95% CI (AUC)	P Value
**Albumin**	rAAA	2.71	84.6%	67.4%	0.816	0.712-0.921	<0.001
**Serum Total Protein**	rAAA	5.21	91.9%	95.2%	0.979	0.955-0.999	<0.001
**PNI**	rAAA	27.11	84.6%	69.6%	0.816	0.714-0.922	<0.001
**CONUT Score**	rAAA	4.50	73.9%	65.4%	0.766	0.658-0.875	<0.001
**Albumin**	30-day mortality	2.35	89.5%	58.8%	0.778	0.666-0.890	<0.001
**Serum Total Protein**	30-day mortality	4.35	93.5%	69.7%	0.827	0.724-0.930	<0.001

**PNI**	30-day mortality	27.11	73.7%	76.5%	0.778	0.666-0.890	<0.001
**CONUT Score**	30-day mortality	5.50	67.6%	63.2%	0.657	0.528-0.785	0.017

Supplementary Table 3. Correlations among anthropometric measures, baseline values of hemoglobin and creatinine, inflammatory biomarkers, and nutritional biomarkers.

Variables		BMI*	Hemoglobin	Creatinine	Albumin	Serum Total Protein	NLR	PNI	CONUT Score
BMI*	Coeficient Correlation	-	-0.056	0.185	0.358	0.155	0.140	0.351	-0.243
	P Value	-	0.623	0.102	**0.012**	0.281	0.217	**0.014**	0.096
Hemoglobin	Coeficient Correlation	-0.056	-	-0.323	0.485	0.657	-0.398	0.488	-0.422
	P Value	0.623	-	**<0.001**	**<0.001**	**<0.001**	**<0.001**	**<0.001**	**<0.001**
Creatinine	Coeficient Correlation	0.185	-0.323	-	-0.210	-0.398	0.263	-0.213	0.207
	P Value	0.102	**<0.001**	-	0.078	**<0.001**	**0.003**	0.075	0.083
Albumin	Coeficient Correlation	0.358	0.485	-0.210	-	0.628	-0.287	0.999	-0.803
	P Value	**0.012**	**<0.001**	0.078	-	**<0.001**	**0.015**	**<0.001**	**<0.001**
Serum Total Protein	Coeficient Correlation	0.155	0.657	-0.398	0.628	-	-0.444	0.626	-0.529
	P Value	0.281	**<0.001**	**<0.001**	**<0.001**	-	**<0.001**	**<0.001**	**<0.001**
NLR	Coeficient Correlation	0.140	-0.398	0.263	-0.287	-0.444	-	-0.300	0.539
	P Value	0.217	**<0.001**	**0.003**	**0.015**	**<0.001**	-	**0.011**	**<0.001**
PNI	Coeficient Correlation	0.351	0.488	-0.213	0.999	0.626	-0.300	-	-0.812
	P Value	**0.014**	**<0.001**	0.075	**<0.001**	**<0.001**	**0.011**	-	**<0.001**
CONUT Score	Coeficient Correlation	-0.243	-0.422	0.207	-0.803	-0.529	0.539	-0.812	-
	P Value	0.096	**<0.001**	0.083	**<0.001**	**<0.001**	**<0.001**	**<0.001**	-
